# Functional Profiling Reveals Critical Role for miRNA in Differentiation of Human Mesenchymal Stem Cells

**DOI:** 10.1371/journal.pone.0005605

**Published:** 2009-05-19

**Authors:** Angela Schoolmeesters, Teresa Eklund, Devin Leake, Annaleen Vermeulen, Queta Smith, Shelley Force Aldred, Yuriy Fedorov

**Affiliations:** 1 Thermo Fisher Scientific, Lafayette, Colorado, United States of America; 2 SwitchGear Genomics, Menlo Park, California, United States of America; Universidad Europea de Madrid, Spain

## Abstract

**Background:**

Mesenchymal stem (MS) cells are excellent candidates for cell-based therapeutic strategies to regenerate injured tissue. Although human MS cells can be isolated from bone marrow and directed to differentiate by means of an osteogenic pathway, the regulation of cell-fate determination is not well understood. Recent reports identify critical roles for microRNAs (miRNAs), regulators of gene expression either by inhibiting the translation or by stimulating the degradation of target mRNAs.

**Methodology/Principal Findings:**

In this study, we employed a library of miRNA inhibitors to evaluate the role of miRNAs in early osteogenic differentiation of human MS cells. We discovered that miR-148b, -27a and -489 are essential for the regulation of osteogenesis: miR-27a and miR-489 down-regulate while miR-148b up-regulates differentiation. Modulation of these miRNAs induced osteogenesis in the absence of other external differentiation cues and restored osteogenic potential in high passage number human MS cells.

**Conclusions/Significance:**

Overall, we have demonstrated the utility of the functional profiling strategy for unraveling complex miRNA pathways. Our findings indicate that miRNAs regulate early osteogenic differentiation in human MS cells: miR-148b, -27a, and -489 were found to play a critical role in osteogenesis.

## Introduction

Human mesenchymal stem cells (hMSC) are non-hematopoietic stromal cells that exhibit multi-lineage differentiation capacity. Adult bone marrow-derived hMSC are easily isolated and expanded in culture. These cells can be differentiated to form a variety of tissues including bone, cartilage, tendon, adipose and other tissues [1], [2]. Tissue specific differentiation of hMSC is a multi-stage process where each step is typically associated with expression of specific markers. In particular, early osteogenesis is accompanied by an increase in bone-specific alkaline phosphatase (AP) activity and by induction of specific biomarkers including SPP1 (osteopontin) among others [3], [4]. Despite the progress made in characterizing hMSC differentiation, the molecular determinants that regulate osteogenesis are not completely understood.

MicroRNAs (miRNAs) modulate gene expression by inhibiting the translation or promoting the degradation of target mRNAs. To date, hundreds of human miRNAs have been identified through experimentation or by *in silico* analyses [5], [6]. Since miRNAs can regulate more than one target, estimates indicate that they regulate up to 30 percent of the protein-coding genes in the human genome, highlighting their importance as regulators of gene expression. Recent studies indicate that miRNAs are involved in the determination of various cell fates – neuronal, muscle and others [7]–[9]. With regard to hMSC, little is known about the role of miRNAs in differentiation [10], [11] or regulation of osteogenesis [12].

Current methodologies for identifying miRNAs focus on measuring levels of miRNAs within a cell type. Unfortunately, expression of miRNAs vary dramatically within cells, often being limited to just a few copies per cell making detection very difficult [13]. These assays correlate the presence of a given miRNA with a specific outcome (i.e. osteogenesis) but fail to identify the role or target(s) of the miRNA. Alternatively, a functional analysis of miRNAs' role in osteogenesis can be obtained by screening a collection of miRNA inhibitors that modulate the endogenous levels of miRNAs during osteogensis in hMSCs. In this study, we found that hsa-miR-148b, -27a and -489 were capable of regulating osteogenic cell fates. Moreover, modulation of the miRNA levels induces differentiation in the absence of external cues and stimulates osteogenesis in over-propagated hMSC. We performed preliminary studies using siRNA-mediated knockdown to identify actual miRNA targets from a subset of *in silico* predicted candidates. Further studies will help define whether these mRNAs play direct or indirect roles in the osteogenic pathway. Together, the data suggests that miRNAs are essential regulators of early osteogenic differentiation of hMSC.

## Results

**Figure 1:**
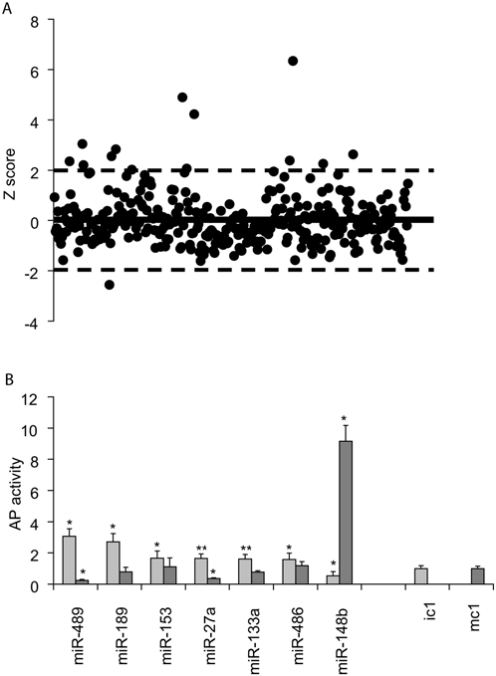
Inhibition of miRNAs dramatically affect osteogenic differentiation of hMSC. (A) Fifteen miRNA inhibitors were selected in the primary screen for their effect on AP activity in differentiated hMSC. Data are representative of a single screen performed in triplicate. (B) Seven inhibitors were confirmed as hits in separate experiments. miRNA mimics for four miRNAs demonstrated no effect on AP activity, -miR-189, -153, -133a and -486. For three other miRNAs, miR -148b, -27a and -489, mimics had an effect that was opposite to one of the corresponding inhibitors. Data are representative of three independent experiments performed in triplicate. (mean+/−SD). *, **: Student's ttest p value between treated cells and corresponding control group, * - p<0.01, ** - p<0.05.

It has been reported that antisense inhibition of specific miRNAs can dramatically affect cellular differentiation [7]–[9]. To determine if miRNAs regulate hMSC osteogenic differentiation, we conducted a functional miRNA screen. hMSC were transfected with a library of miRNA inhibitors and subjected to osteogenic differentiation by incubation in differentiation media. Early osteogenesis in hMS cells is accompanied by the differential expression of multiple markers of osteogenic differentiation. One such example is a significant increase in alkaline phosphatase (AP) activity making this a convenient marker for osteogenic differentiation [4]. In our experiments a change in AP activity in transfected cultures was used as a marker of osteogenic differentiation. Candidate miRNAs were identified as described in Materials and Methods. Briefly, Z-score values for an AP activity assay were used to select candidates from the initial functional miRNA screen. miRNA inhibitors that demonstrated Z-score values that were two standard deviations (SD) from the control were selected as hits and were included in follow-up experiments (Fig. 1a).Of 396 miRNA inhibitors, fifteen were identified in the primary screen for their effect on differentiation. Seven inhibitors were subsequently confirmed in independent experiments (Fig. 1b); six inhibitors (miR-489,-189,-153,-27a,-133a,-and -486) increased AP activity and one (hsa-miR-148b) decreased activity in differentiated hMSC. An increase in AP activity upon inhibition of a miRNA indicates that the miRNA may be necessary for suppression of differentiation. In contrast, a decrease in AP activity suggests that the miRNA may be necessary for stimulation of osteogenesis. To investigate if miRNA activity is sufficient for regulation of early osteogenic differentiation, we initiated experiments with miRNA mimics. Change in AP activity from cells transfected with mimics would indicate that miRNA activity is sufficient to stimulate osteogenesis. Four of the miRNA mimics demonstrated no effect on AP activity (Fig. 1b), miR-189, -153, -133a and -486, suggesting that these miRNAs while necessary are not sufficient for regulation of early osteogenesis in hMSC under the effect of external stimuli. Three miRNA mimics, miR-148b, -27a and -489, had an effect that was opposite to their respective inhibitors (Fig. 1b). For all three miRNA inhibitors and mimics, osteogenesis was affected in a concentration dependent manner (Fig. S1). All together, these findings demonstrate that these three miRNA are both necessary and sufficient to modulate early osteogenesis in hMSC: miR-489 and miR-27a down-regulate and miR-148b up-regulates differentiation.

miR-148b, -27a and -489 were found to be essential for osteogenesis in our experiments. We hypothesize that their functions may correlate with the level of expression in hMSC under propagation or differentiation conditions. To study expression of these miRNAs that are essential for osteogenesis we employed RT-PCR-based expression analysis. The analysis revealed that miR-148b and miR-27a both can be detected in undifferentiated and differentiated cells (Table S1.) at the level of tens and hundreds of copies per cell, respectively. For comparison, miR-16, a miRNA that was reported to be ubiquitously expressed in thousands of copies per cell [13] was detected at nearly 8000 copies per cell for both undifferentiated and differentiated hMSC (data not show).

We found that upon osteogenic differentiation, expression of miR-27a decreases 41% (p-value<0.01) and miR-148b increases 67% (p-value<0.02) (Table S1). Expression of miR-489 was detected at very low levels in hMSC. Only 0.9 copies per cell were found in cells harvested under propagation conditions. After induction of differentiation, expression of miR-489 becomes almost undetectable, decreasing 10-fold (p-value<0.1) (Table S1). Overall, this data indicates that miR-148b, -27a and -489 expression levels correlate with their function. In general, miRNAs that stimulate differentiation demonstrated higher expression in differentiated cells, and miRNAs that mitigate differentiation demonstrated lower expression in differentiated cells.

Our study revealed that miR-148b, -27a and -489 play critical roles in osteogenesis in hMCS. Osteogenesis is only one of several differentiation pathways that hMSC can undergo. Thus, miRNA modulation may be either specific or non-specific to osteogenic differentiation. To test specificity of the effect, we examined the ability of transfected hMSC to undergo adipogenic differentiation. Transfection with miR-148b, -27a and -489 inhibitors produced no change in number of adipocytes in hMSC cultures (Fig. S2). This demonstrates that modulation of miR-148b, -27a and -489 levels has no effect on hMSC adipogenesis and suggests that the miRNA effect may be specific.

**Figure 2:**
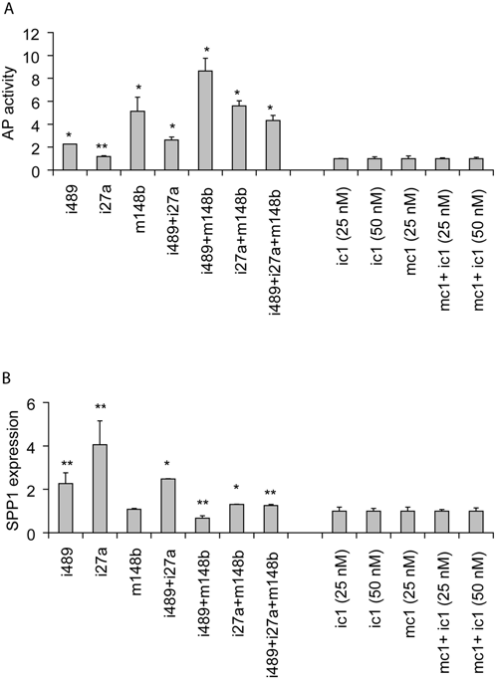
Adjustment of the miRNAs activity alone induced osteogenic differentiation of hMSC in the absence of other differentiation stimuli. Cells transfected with miRNA inhibitors or mimics alone or in combination, demonstrated drastic increase in total AP activity (A), and up-regulation of SPP1, another marker of early ostogenesis (B) . Data are representative of three independent experiments performed in triplicate. (mean+/−SD). *, **: Student's ttest p value between treated cells and corresponding control group, * - p<0.01, ** - p<0.05.

Osteogenic differentiation of hMSC is achieved by incubating in the presence of a differentiation cocktail, including dexamethasone, ascorbate and β-glycerophosphate [4]. Our data from experiments with differentiated hMSC suggests that the modulation of miR-148b, -27a and -489 may be sufficient for the induction of osteogenesis. To test this hypothesis, hMSC were incubated in propagation media lacking differentiation stimuli and miRNA levels were altered. In these experiments, alteration of the miRNA levels alone induced osteogenic differentiation of hMSC in the absence of other differentiation stimuli (Fig. 2, Fig. S3). Cells transfected with miR-489 inhibitor or miR-148b mimic alone or in combination demonstrated a drastic increase in total AP activity and in the number of AP-positive cells (Fig. 2a, Fig.S3). Up-regulation of another marker of early osteogenic differentiation, SPP1 (Fig. 2b), was also found in transfected cells. Interestingly, while inhibitor miR-27a was able to up-regulate osteogenesis in transfected cells incubated in differentiation media, it demonstrated very low effect on cells in propagation media (Fig. 2a). Also, induction of SPP1 expression was found in cells transfected with miR-27a inhibitor (Fig. 2b). These findings indicate that miR-27a and -489 may affect both related and unrelated pathways.

hMSC can originate from donors with variable genetic background and certain donor-to-donor variability has been reported [14]. Also, human adult stem cells with osteogenic potential can be derived from various tissues, including adipose tissue [2]. In our experiments, we found that miRNA modulators were effective in cells derived from two independent donors, male and female (Fig. S4a). Furthermore, miRIDIAN miRNA modulators were found to be successful in stimulation of osteogenesis in adult stem cells derived from adipose tissue (Fig. S4b and S4c).

**Figure 3:**
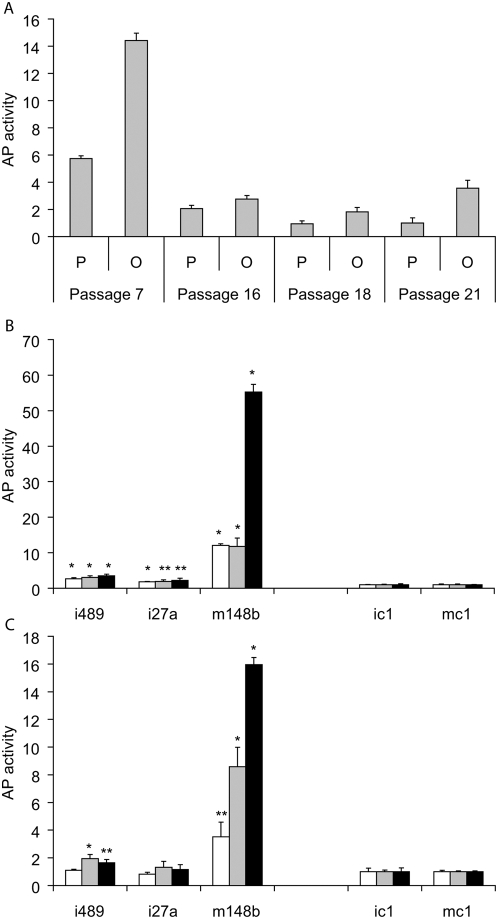
Alteration of the miRNAs activity rescues osteogenic potential in transfected hMSC with high passage number. (A) Bone marrow hMSC with high passage number demonstrate a decrease in basal level of AP activity and poor response to differentiation media. hMSC were plated in 96-well plates and incubated either in propagation (P, grey bar) or osteogenic (O, black bar) media for 6 days. Data normalized to relative AP activity values obtained from passage 21 hMSC incubated in propagation media. (B and C) Transfection with miRNA inhibitors and mimics restore differentiation in over-propagated hMSC incubated in propagation (B) or differentiation (C) media. hMSC propagated for certain number of passages (16, white bar, 18, grey bar or 21 , black bar) plated in 96-well plates and transfected with inhibitors and mimics (as indicated, all at 25 nM). Data are representative of three independent experiments performed in triplicate. (mean+/−SD). *, **: Student's ttest p value between treated cells and corresponding control group, * - p<0.01, ** - p<0.05.

To generate an amount of material that is necessary for tissue replacement therapy, hMSC can be subjected to propagation *in vitro*. Unfortunately, hMSC that are extensively propagated tend to loose their differentiation potential [14]–[16]. In our experiments, bone marrow hMSC with high passage number demonstrated decreased basal level of AP activity and poor response to differentiation media (Fig. 3a). Surprisingly, transfection with miR-148b mimic, miR-489 or -27a inhibitors were able to rescue osteogenic potential in hMSC with high passage number. Transfection with miRNA modulators induced total AP activity in high passage number hMSC (Fig. 3b, c). Expression of SPP1 was also up-regulated in hMSC with high passage number transfected with miRNA activity modulators (Fig. S5 a,b).

As determined by the miRanda 3.0 algorithm and L2L gene ontology tool [17], both miR-27a and -489 target a number of genes that are related to bone remodeling and skeletal development (Table S2). Three genes were present in both gene lists, AHSG, PEX7 and CHRD. Products of AHSG and CHRD encode Bone Morphogenetic Protein (BMP) signaling inhibitors [18], [19]. Similarly, PEX7 is a gene which encodes the receptor for a class of peroxisomal matrix enzymes, critical enzymes in bone morphogenesis [20]. RT-PCR-based study of gene expression demonstrated that both CHRD and PEX7 mRNA are present in hMSC but only PEX7 is regulated by miR-489 and -27a (Fig. S6a). Expression of AHSG was not detected in hMSC by RT-PCR (data not shown). We hypothesize that if PEX7 is a key target of miR-27a and miR-489 then its knockdown may mimic effect of these miRNAs and inhibit osteogenic differentiation. Experiments with siRNA-mediated knockdown of PEX7 did not result in a significant decrease of AP activity in hMSC under differentiation conditions (Fig. S6b). Thus, while PEX7 still can be a target of miR-27a and miR-489 it may not mediate effect of these miRNAs on osteogeneis.

**Figure 4:**
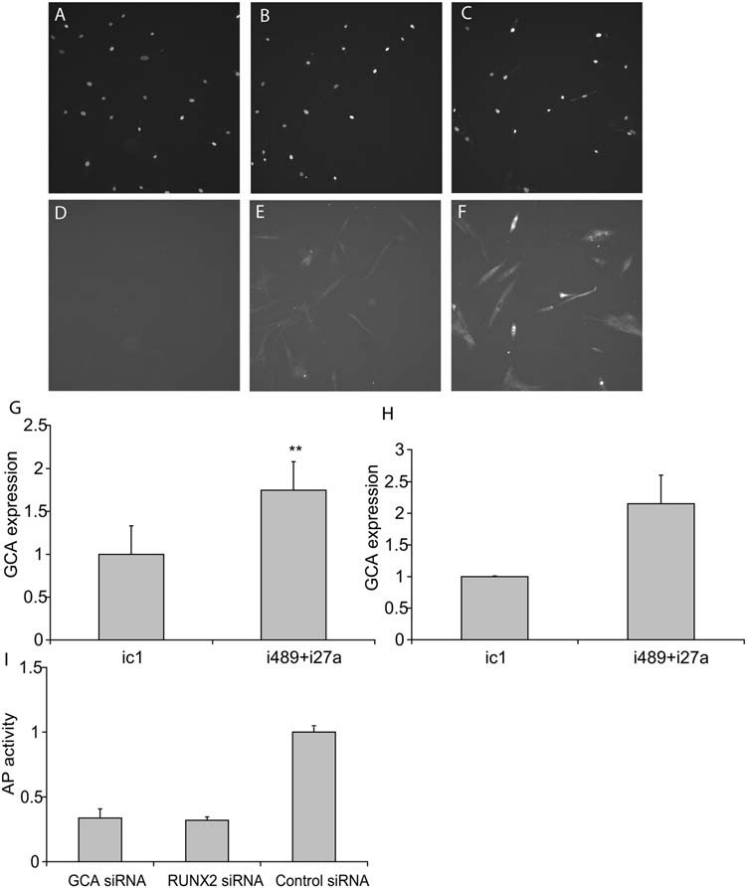
Negative effect of miRNAs, miR-489 and -27a, on osteogenesis in hMSC is, at least in part, mediated by repression of GCA gene expression. (A–G) Transfection with inhibitors of miR-489 and -27a results in up-regulation of GCA protein expression. (A–F). hMSC were transfected with control molecule, ic1 (25 nM) or with combination of inhibitors, i489+i27a (12.5 nM each). GCA protein expression was determined by immunofluorescence as described in Materials and Methods. Cells were stained with Hoechst 33342 (A–C) and GCA specific primary antibody, followed by a secondary antibody (E,F). Control wells were treated with secondary antibody only and demonstrate no specific staining (D). (G) Quantitation of GCA immunohystochemistry. Data shown represents three independent transfections, four fields each. (mean+/−SD). ** - Student's ttest p value between treated cells and corresponding control group, p<0.05. (H) RT-PCR-based study of gene expression demonstrated that the expression of GCA mRNA is regulated by miR-489 and -27a. hMSC were transfected with Inhibitor Control molecule 1 (IC1, 25 nM) or with a combination of inhibitors for miR-27 and -489 (i27a+i489, 12.5 nM each). Transfected cells were incubated for 2 days in propagation media and then harvested for totat RNA. (I) siRNA-mediated knockdown of GCA (grancalcin) resulted in a significant decrease of AP activity in hMSC under differentiation conditions. hMSC were transfected with Control siRNA, RUNX2 and GCA siRNA (each at 50 nM). Cells were switched to osteogenic media 24 hr after transfection and harvested 6 days after the switch. Data shown represents two independent transfection experiments performed in duplicate (H) or triplicate (I) (mean+/−SD). **: Student's ttest p value between treated cells and corresponding control group, p<0.05.

miR-489 and -27a have 80 predicted targets in common (Table S3). Our preliminary analysis of siRNA mediated knockdown of target genes that are not related to osteogenesis revealed that some of these genes may be involved in the regulation of osteogenesis in hMSC (Fig. 4 and data not shown). While a control siRNA has no effect on osteogenesis, siRNA-mediated knockdown of GCA (grancalcin) or SLC22A2 (solute carrier protein) did result in a significant decrease of AP activity in hMSC under differentiation conditions (Fig. 4i, and data not shown). Expression of SLC22A2 was not detected by RT-PCR in hMSCs used in our study. In a contrary, both RT-PCR-based study and immunohistochemistry-based analysis of gene expression demonstrated that GCA mRNA is present in hMSC and regulated by miR-489 and -27a (Fig. 4). Additionally, we found that GCA 3′UTR reporter activity is regulated by miR-489 and -27a (Fig. S7). While further studies are needed to investigate the possible roles of miR-27a and -489 targets in greater detail, our data suggests that the inhibitory effect of miR-489 and -27a is, at least in part, mediated by repression of GCA.

## Discussion

Although the biological functions of most miRNA are unknown, their importance for molecular regulation of differentiation, cell proliferation, and cell death has been demonstrated [7]–[9]. By performing a functional miRNA screen, we were able to identify three miRNAs that are critical regulators of early osteogenesis in hMSC, namely miR-148b, -27a and -489.

The candidate miRNAs, miR-489, -27a and -148b, are representatives of three miRNA families. Family members are thought to have similar target specificity [21] and may affect cellular physiology in a common fashion. In human cells, the miR-489 family is represented by a single member, miR-489. The miR-27 family is represented by two members, miR-27a and -27b. Similarly, family miR-148 is represented by miR-148a and 148b. Human miR-27a and -27b differ by a single nucleotide at position 19. miR-148a and -148b differ by two nucleotides at positions 7 and 8. We found that the miR-27b inhibitor induces a relatively small increase in AP activity in hMSC. AP activity for miR-148a inhibitor treated/transfected cells was extremely variable and fell into an outlier category (data not shown). Further experiments demonstrated that miR-148a mimic was able to induce AP activity in hMSC, but it was at least two-fold less potent than the mimic of miR-148b (data not shown). Based on these results both miR-27b and miR-148a were excluded from further study.

Upon differentiation, expression of miR-27a and -148b changed and correlated with the level of osteogenic differentiation that was observed at day six. Expression of miR-489 was detected at a very low level in undifferentiated MS cells and appeared to decrease even further upon differentiation. Our findings agree with previously published data on expression of miR-148b and -489. miR-148b was found among a group of miRNAs that were associated with osteoblast differentiation, in a particular 3D substrate [22]. In contrast, miR-489 was found to be down-regulated under similar conditions [23].

Other groups have reported that miR-26a and miR-125b play an important role in the regulation of osteogenic differentiation in human adipose tissue-derived stem cells and in mouse ST2 cell line respectively [12], [24]. In our screening experiments, inhibitors for both of these miRNAs produced significant variability and were outliers by our criteria. Further studies will be required to determine if these two miRNAs play roles in osteogenic differentiation of human bone marrow MS cells.

miRNAs target a multitude of genes and regulate cellular physiology through different mechanisms. Both miR-489 and -27a down-regulated osteogenesis, suggesting the possibility of common gene targets (Fig. 1b, 2b, Fig. S2). Using miRanda 3.0 an *in silico* prediction tool, over 2,000 genes can be predicted as targets for these miRNAs, with 81 gene targets common for both miRNAs. Analysis of the predicted gene targets revealed that APL, a gene encoding liver/bone/kidney-specific alkaline phosphatase, may be a target for miR-27a but not for miR-489. Similarly, SPP1 may be a target for miR-489 but not for miR-27a. As expected, inhibition of each miRNA induced both markers, respectively. Interestingly, inhibition of miR-489 had a stronger effect on AP activity induction than inhibition of miR-27a. However, inhibition of miR-27a demonstrated more substantial activation of SPP1 expression in comparison with inhibition of miR-489. When either APL or SPP1 were down-regulated using siRNAs, there was no effect on the other gene (data not shown). Together, this data suggests that the two miRNAs regulate certain osteogenesis-inducing genes which are likely to be located upstream of APL and SPP1.

Our findings indicate that miR-27a, -489 and -148b may affect both related and unrelated pathways. Inhibitor of miR-489 was able to stimulate differentiation in either propagation or differentiation conditions while inhibitor of miR-27a was active in the differentiation conditions only. Interestingly, the addition of i27a to the combination i489+m148b (Fig. 2b) was detrimental for osteogenic differentiation in cells under propagation conditions while no change have been expected. One can speculate that possible explanation of this finding may include a cross-talk between multidute of direct and indirect targets that are regulated by all three miRNAs in complex spatio-temporal system of mNSCs differentiation. All together three miRNAs of interest have over 2300 predicted direct targets in partially overlapping sets. Further extencive study will be needed to elucidate all possible interactions between their targets.

*In silico* analysis of predicted targets followed by siRNA-mediated knockdown experiments indicated that GCA (grancalcin) is a regulator of osteogenesis in hMSCs. GCA is a common target for miR-27a and -489 and its expression is directly regulated by these miRNAs (Fig. 4). Downregulation of GCA expression using siRNAs resulted in a drastic decrease in osteogenic differentiation of hMSCs (Fig. 4). Grancalcin, a product of GCA, is a penta-EF-hand Ca2+-binding protein [25], [26] . While some of the members of the PEF-hand protein family, calpains, have been described as potent regulators of differentiation in hMSCs, grancalcin was previously not associated with ostegenesis [27].

Analysis of over 800 genes that are predicted as targets for miR-148b (miRanda 3.0, [28], [29] identified multiple genes that are related to skeletal development ( Table S2). While compelling, further studies will be required to confirm the role of these genes in miRNA-mediated regulation of hMSC differentiation.

In conclusion, we have demonstrated the utility of the functional profiling strategy for unraveling complex pathways. Specifically, we have begun to characterize the distinct regulatory roles for three miRNAs in osteogenesis. miR-148b, -27a, and -489 were found to play a critical role in osteogenesis in human mesenchymal stem cells. Understanding the complex interplay of miRNAs and their targets will aid in developing viable cell-based therapeutic strategies in regenerative medicine.

## Materials and Methods

### Cell propagation and differentiation

hMSC were purchased from Lonza (Bazel, Switzerland, Lot# 4f1301, and # 6f3674) and ScienCell (Carlsbad, CA , lot #1134). Cells were propagated using either hMSC propagation media (Lonza) or DMEM supplemented with 10% FBS (Thermo Fisher Scientific). Cells maintained in either media demonstrated comparable proliferation rates and differentiation capacity toward osteogenic and adipogenic lineages.

Differentiation was induced using specific osteogenic or adipogenic stimulating media (Lonza) according to protocols provided by cell supplier. As indicated by the manufacturer, osteogenic differentiation media was supplemented with dexamethasone, ascorbate and β-glycerophosphate. Induction media for the adipogenic differentiation contained dexametasone, IBMX and insulin, and maintenance media was with insulin only. For osteogenic differentiation, cells were harvested six days after induction.

### Transfection and library screening

Transfection optimization was performed using a siRNA SMARTpool targeting human PPIB (NM_000942, Thermo Scientific, Lafayette, CO) complexed with the lipid-based transfection reagents DharmaFECT 1, 2, 3, or 4. DharmaFECT 1 was selected for all further transfections as it provided over 90% transfection efficiency in both low and high passage number hMSC. Transfection of miRNA inhibitors and mimics was confirmed by the uptake of fluorescently labeled control molecules miRNA Inhibitor Control 1 and Mimic Control 1 (ThermoFisher Scientific, Waltham, MA Fig S8) . RUNX2 (NM_001015051, NM_001024630, NM_004348) siRNA pool and Control Non-Targeting siRNA pool (Thermo Scientific, Waltham, MA ) were used to calculate the Z′ factor for the AP activity assay and to monitor transfection efficiency throughout the whole study. The Z′ factor for a pair of positive and negative controls (RUNX2 and Control siRNA pools) for total AP activity was 0.3 or better indicating that the assay remains robust (data not shown). These and other siRNA pools (GCA (NM_012198) and SLC22A2 (NM_003058)) were used at the concentration of 50 nM.

miRIDIAN miRNA inhibitors and mimics (Thermo Scientific, Waltham, MA) were described previously [30] . In brief, these molecules were designed to target 396 human miRNAs (Sanger miRBase v 8.0) were chemically synthesized and transfected into hMSC using optimized transfection conditions. For transfection, cells were plated into 96-well plates (ThermoFisher Scientific, Waltham, MA) at a density of 2,500 (osteogenic differentiation) or 10,000 (adipogenic differentiation) cells per well. miRIDIAN miRNA Inhibitor Control 1 and Mimic Control 1 (IC1 and MC1, respectively; Thermo Fisher Scientific, Waltham, MA) were used throughout the study as negative control molecules. IC1 and MC1 target and mimic respectively, cel-mir-67, a *C. elegans* miRNA which are expected to have no sequence homology to miRNAs derived from the human genome.

Preliminary experiments with the IC1 and MC1 control molecules demonstrated that for both control molecules, 25 nM was the highest concentration that produced no effect on AP activity in differentiating hMSC (Fig. S9a). Thus, a concentration of 25 nM was selected for the majority of experiments with miRNA inhibitors and mimics. In all experiments, relative AP activity was normalized to IC1 or MC1 controls for each plate separately. Change in AP activity in transfected cultures was used as a measure of osteogenic differentiation to monitor effect of miRNA inhibitors. Screening experiments were performed in triplicate and means, standard deviations, and CVs were determined. Inhibitors with CVs more or equal to 0.5 were discarded as outliers.

Z-score values for the AP activity assay were used to select hits in the initial functional miRNA profiling experiments. Z-score was calculated as follows: 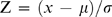
where: *x* is a raw score to be standardized, *σ* is the standard deviation of the population, and *μ* is the mean of the population. Population was comprised of means from the entire screening experiment, excluding controls. miRNA inhibitors that demonstrated Z-score values that were outside of 2SD window were selected as hits and were subjected to hit validation experiments (Fig. 1a). For a MIARE compliant version of the miRNA inhibitor screen data please contact corresponding author at Yuriy.Fedorov@thermofisher.com.

### Differentiation assays

In our experiments, we found that under the effect of exogenous stimuli, hMSC undergo osteogenic differentiation. By day six following treatment with differentiation media, total AP activity increased two to three-fold (Fig. S9b) and the fraction of AP-positive cells increased up to nine-fold (Fig. S9c). In addition to induction of AP activity, early osteogenesis is associated with stimulation of specific biomarker expression, including SPP1, a gene that encodes osteopontin [3]. By day six, SPP1 increased more than six-fold over control cells that were maintained in propagation media (Fig. S9d).

In our experiments, cells were assayed for osteogenic differentiation at day six. Total AP activity was determined by p-nitrophenol phosphate conversion method using an AP activity kit (BRSC, NY). Raw AP activity values were normalized to total protein determined by Advanced Protein Assay (Cytoskeleton, CO). To determine the fraction of AP-positive cells in the hMSC cultures, cells were fixed in 4% paraformaldehyde (EM Sciences,Hatfield,PA) and stained with ELF97 kit (ATCC, MD). SYTO Green (Life Technologies, Carlsbad, CA) was used as a nuclear counter stain. Adipogenic differentiation was assayed after incubation in induction media for 6 days and 3 days in maintenance media. Adipogenesis was determined by Oil Red O staining (Sigma, St.Louis, MO) combined with H33342 nuclear dye (Life Technologies, Carlsbad, CA). Both ELF97 and Oil Red O images of stained cells were acquired using an ArrayScan VTi (Thermo Fisher Scientific, Waltham, MA) and quantified using the Target Activation Bioapplication software tools (Thermo Fisher Scientific, Waltham, MA).

### miRNA expression analysis

The RNeasy Plus kit (Qiagen, Valencia, CA) was used for total RNA purification. miRNA expression analysis was performed using quantitative PCR. RNA was harvested from hMSC with an average yield of 3 pg/cell. The miRNA copy number per cell was estimated based on standard curves of synthetic miRNAs. Total RNA was added to RT reaction in an amount of 120 ng. TaqMan® MicroRNA Reverse Transcription Kit (Life Technologies, Carlsbad, CA) was used for the RT step. Corresponding TaqMan® Human MicroRNA Assays (Life Technologies, Carlsbad, CA) were used to determine the copy number of miR- 498, -27a, and -148b. The ubiquitously expressed miRNA, hsa-miR-16 was used as a positive control. Cel-miR-67 was used as a negative control. Reactions were set up according to the supplier's protocol. Additionally, all sets of primers were tested with synthetic RNA corresponding to the miRNAs. Absolute quantification values were acquired using the Stratagene Mx3005p.

### Predicted miRNA target analysis

miRBase Targets Release Version 2 (Wellcome Trust Sanger Institute) was used to search for predicted targets. miRanda 3.0 algorithm was used for target prediction. Predicted target lists were analyzed for association with Gene Ontology (GO) terms using L2L Microarray Analysis Tool (University of Washington) [17].

### Ananlysis of miRNA activity using 3′UTR reporter system

The effects of selected synthetic miRNA mimics on endogenous human 3′UTRs were measured using reporter constructs (SwitchGear Genomics, Menlo Park, CA). Briefly, 3′UTR-reporter vectors were constructed by cloning UTRs from two known human UTRs and two random genomic fragments downstream of a firefly luciferase (luc2P) reporter cassette (Promega, Madison, WI). Genomic coordinates of the cloned fragments described in Table S4. Transcription of the hybrid transcripts was driven by the constitutively active RPL13 promoter on every vector. A control vector containing no extra UTR sequence and the two vectors containing random genomic sequence were used to control for overall signal variation based on the non-specific response of cells to different treatments.

Transient transfection assays were conducted in HT1080 human fibrosarcoma cells (ATCC, Manassas, VA) in 96-well plates. Briefly, 6,000 cells per well were seeded in culture medium for 24 hr before transfection. Next, in each well, 0.15 uL Dharmafect Duo (Thermo Fisher Scientific, Waltham, MA) was used to transfect 100 ng of plasmid DNA and either a miRNA mimic or a non-targeting control according to manufacturer's protocol. Mimics and non-targeting controls were added to yield final concentrations of 12.5 nM. After 24 hours, 100 uL of Steady-Glo (Promega, Madison, WI) was added into each well, incubated at room temperature for 30 min, and then signals were read in a standard plate luminometer (Molecular Devices, Sunnyvale, CA). Each transfection was conducted and assayed in triplicate.

After normalizing for between-treatment signal variation, a Student's t-test (p<0.05) was used to assess whether the activity of the cloned UTR fragment was different in the presence of a miRNA mimic as compared to the non-targeting control. Finally, a log2 ratio of activity was calculated for miRNA mimic treated/non-targeting control signals.

### Gene expression analysis

PPIB (NM_000942), RUNX2 (NM_001015051, NM_001024630, NM_004348) and SPP1 (NM_000582, osteopontin) expression was determined using branched DNA assay (Panomics, Fremont, CA). GAPDH was used as a housekeeping reference gene for normalization purposes. In all experiments, relative gene expression was normalized to IC1 or MC1 controls for each plate separately.

Expression of GCA (NM_012198), SLC22A2 (NM_003058), PEX7 (NM_000288.3), AHSG (NM_001622), and CHRD (NM_003741) was determined by qRT-PCR. Corresponding TaqMan® Gene Expression Assays were purchased from Life Technologies (Carlsbad, CA) - GCA Assay ID Hs00201854_m1, SLC22A2 Assay ID Hs00533907_m1, PEX7 Assay ID Hs00165464_m1, AHSG Assay ID Hs00155659_m1, and CHRD Assay ID Hs00415315_m1. High-Capacity cDNA Reverse Transcription Kit was used for the cDNA synthesis step. Human GAPD TaqMan® Endogenous Control (NM_002046.3) was used to determine GAPD expression for analysis of relative expression of the specific genes. Reactions were set up according to the supplier's protocol. The qPCR step and the Ct values that were used for further analysis were obtained using the Stratagene Mx3005p.

GCA (NM_012198) protein expression was determined by immunofluorescence. Cells were fixed day six after transfection with 4% paraformaldehyde (EM Sciences, Hatfield, PA). Cell plates were blocked with buffer containing 1% BSA combined with H33342 nuclear dye (Life Technologies, Carlsbad, CA). Primary anti-GCA mouse monoclonal antibody (H00025801-M01) was purchased from Abnova Corporation (Taiwan). The GCA antibody was diluted 1 to 25 for a final concentration of 40μg/mL. Secondary antibody, Goat Anti-Mouse IgG (H+L) DyLight 649 conjugated (Thermo Fisher Scientific, Waltham, MA), was used at a concentration of 2μg/mL, a 1 to 500 dilution. Images of stained cells were acquired using an ArrayScan VTi (Thermo Fisher Scientific, Waltham, MA) and quantified using the Target Activation Bioapplication software tools (Thermo Fisher Scientific, Waltham, MA).

### Statistical Analysis

Student's t-test was used to evaluate statistical significance.

## Supporting Information

Figure S1 Both inhibitors and mimics affect osteogenesis in a concentration dependent manner. hMSC were transfected with miRNA inhibitors (A) and mimics (B) as indicated. Transfected cells were switched to differentiation 24 hr after transfection. AP activity was measured in hMSCs incubated in osteogenic media for 6 days (transfected cells and untransfected control cells, UNTR/Diff) or in untransfected cells incubated in propagation media (UNTR/Undiff). Data are representative of two independent experiments performed in triplicate. (mean+/−stdev).
(3.16 MB TIF)

Figure S2 Inhibition of miR-148b, -27a and -489 does not influence adipogenesis in hMSC. hMSC were transfected with miRNA inhibitors or control molecules ( all at 25 nM). Transfected cells were switched to differentiation 24 hr after transfection. Adipogenesis was determined as a fraction (%) of adipocytes in cultures. Data are representative of three independent experiments performed in triplicate. (mean+/−stdev).
(1.55 MB TIF)

Figure S3 Alteration in miRNA activity results in increase of osteogenesis as measured by number of AP-positive cells. (A–G) Effect of alteration in miRNA activity results in increase of osteogenesis as measured by number of AP-positive cells in hMSC cultures. UNTR/Diff - untransfected differentiated cells, UNTR/Prop - untransfected undifferentiated cells, IC1,- cells transfected with Inhibitor Control Molecule 1, MC1,- cells transfected with Mimic Control Molecule 1, i27a - cells transfected with Inhibitor for miR27a, i489 - cells transfected with Inhibitor for miR489, m148b - cells transfected with mimic for miR148b. (H) Quantitative analysis of results obtained in experiments partially depicted in (A–F).
(9.74 MB TIF)

Figure S4 Alteration in miRNA activity produced similar results in hMSCs from two different donors and in human adult stem cells originated from adipose tissue. (A) Results obtained on AP activity of hMSCs from two different donors Alteration in miRNA activity in human adult stem cells derived from adipogenic tissue results in increase of AP activity (B) and up-regulation of SPP1 expression (C). Cells were treated as described in Supplemental Figure 2 legend are representative of three independent experiments performed in triplicate. (mean+/−stdev).
(8.66 MB TIF)

Figure S5 Alteration of the miRNAs activities transfection rescues osteogenic potential in transfected hMSC with high passage number. Transfection with miRNA inhibitors and mimics restores differentiation in overpropagated hMSC incubated in propagation (A) or differentiation (B) media as measured by SPP1 (osteopontin) expression. Data are representative of three independent experiments performed in triplicate. (mean+/−stdev).
(3.16 MB TIF)

Figure S6 Effect of on osteogenesis miR-489 and -27a is not mediated by repression of CHRD or PEX7. (A) RT-PCR-based study of gene expression demonstrated that the expression of PEX7 but not CHRD mRNA is regulated by miR-489 and -27a. (A) siRNA-mediated knockdown of PEX7 did not produce a significant decrease of AP activity in hMSC under differentiation conditions. Data shown represents two independent transfection experiments performed in duplicate (A) or triplicate (B) (mean+/−SD).
(3.16 MB TIF)

Figure S7 GCA and PEX7 3′UTR reporter activity is regulated by miR-489 and -27a. HT1080 cells were transfected with Mimic Control molecule 1 (mc1, 12.5 nM) or with a combination of inhibitors for miR-27 and -489 (i27a+i489, 6.25 nM each) and with 3′UTR reporter constructs as imdicated. Transfected cells were incubated for 48 and then harvested and processed as described in Materials and Methods. Data shown represents 3 individual transfections (mean+/−SD). **- p<0.05, Student's ttest p value between treated cells and corresponding control group.
(0.87 MB TIF)

Figure S8 Uptake of fluorescently labeled Inhibitor Control Molecule 1 or Mimic Control Molecule 1 by hMSCs. Cells were transfected with Dy549-labeled MimicControl molecule 1 (C, D) or Inhibitor Control Molecule 1 (A,B) as described in Materials and Methods. Live cells were stained with Hoechst 33342 nuclear dye (A,C) 24 hr after transfection and photographed.
(3.14 MB TIF)

Figure S9 Osteogenic differentiation of hMSCs (A) Activation of AP in hMSCs incubated in osteogenic media for 6 days. Cells were incubated either in propagation media (Propagation) or in osteogenic media (Differentiation). (B) Increase in the number of AP-positive cells in differentiated cultures of hMSC harvested at 6 days of incubation in osteogenic media. Cells were incubated either in propagation media (Propagation) or in osteogenic media (Differentiation). (C) Upregulation of SPP1 expression in hMSCs incubated in osteogenic media for 6 days. Cells were incubated either in propagation media (Propagation) or in osteogenic media (Differentiation). (D) Effect of control miRNA inhibitor and mimic on differentiation of hMSCs. Cells were transfected with MimicControl molecule 1 (MC1) or Inhibitor Control Molecule 1 (IC1) as indicated. Transfected cells were switched to differentiation 24 hr after transfection. AP activity was measured in hMSCs incubated in osteogenic media for 6 days (MC1, IC1 and UNTR/Diff - untransfected control cells) or in untransfected cells incubated in propagation media (UNTR/Undiff) . Cells were incubated either in propagation media (Propagation) or in osteogenic media (Differentiation). Data are representative of three independent experiments performed in triplicate. (mean+/−stdev).
(2.89 MB TIF)

Table S1 Expression of miR-148b, -27a and -489 in hMSCs under propagation and differentiation conditions.
(0.05 MB DOC)

Table S2 Genes that are predicted as a target for miR-148b, 27a and 489 and involved into skeletal development.
(0.08 MB DOC)

Table S3 Genes that are predicted as common targets for miR-27a and -489.
(0.09 MB DOC)

Table S4 Genomic coordinates of the fragments cloned into 3′UTR reporter constructs.
(0.04 MB DOC)
