# Supplementary figures and images for: Functional Profiling Reveals Critical Role for miRNA in Differentiation of Human Mesenchymal Stem Cells
Source: PLoS One. 2009 May 19;4(5):e5605. doi: 10.1371/journal.pone.0005605 (PMC2680014; doi:10.1371/journal.pone.0005605)

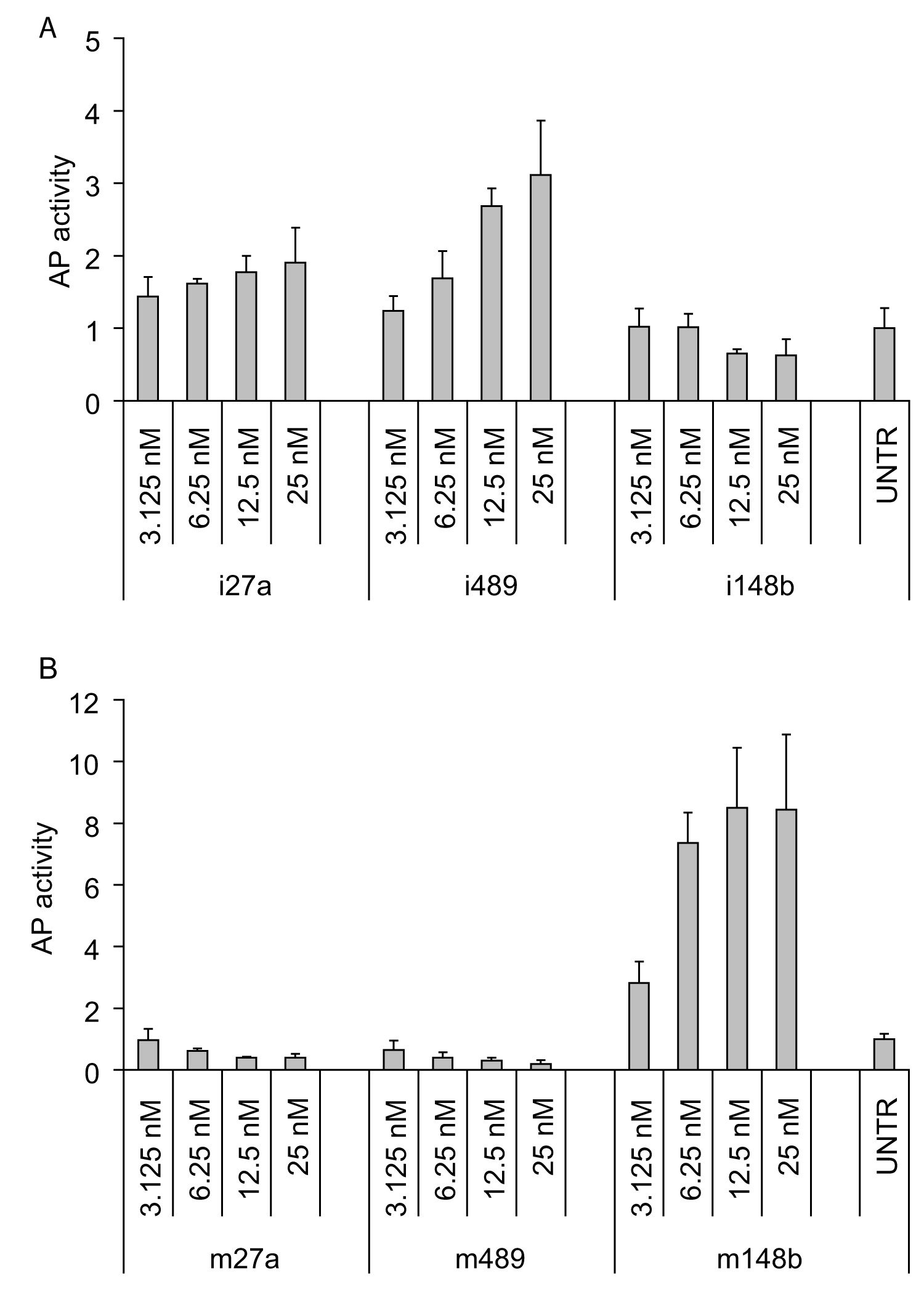

Supplement: Figure S1 — Both inhibitors and mimics affect osteogenesis in a concentration dependent manner. hMSC were transfected with miRNA inhibitors (A) and mimics (B) as indicated. Transfected cells were switched to differentiation 24 hr after transfection. AP activity was measured in hMSCs incubated in osteogenic media for 6 days (transfected cells and untransfected control cells, UNTR/Diff) or in untransfected cells incubated in propagation media (UNTR/Undiff). Data are representative of two independent experiments performed in triplicate. (mean+/−stdev). (3.16 MB TIF) [file pone.0005605.s001.tif]

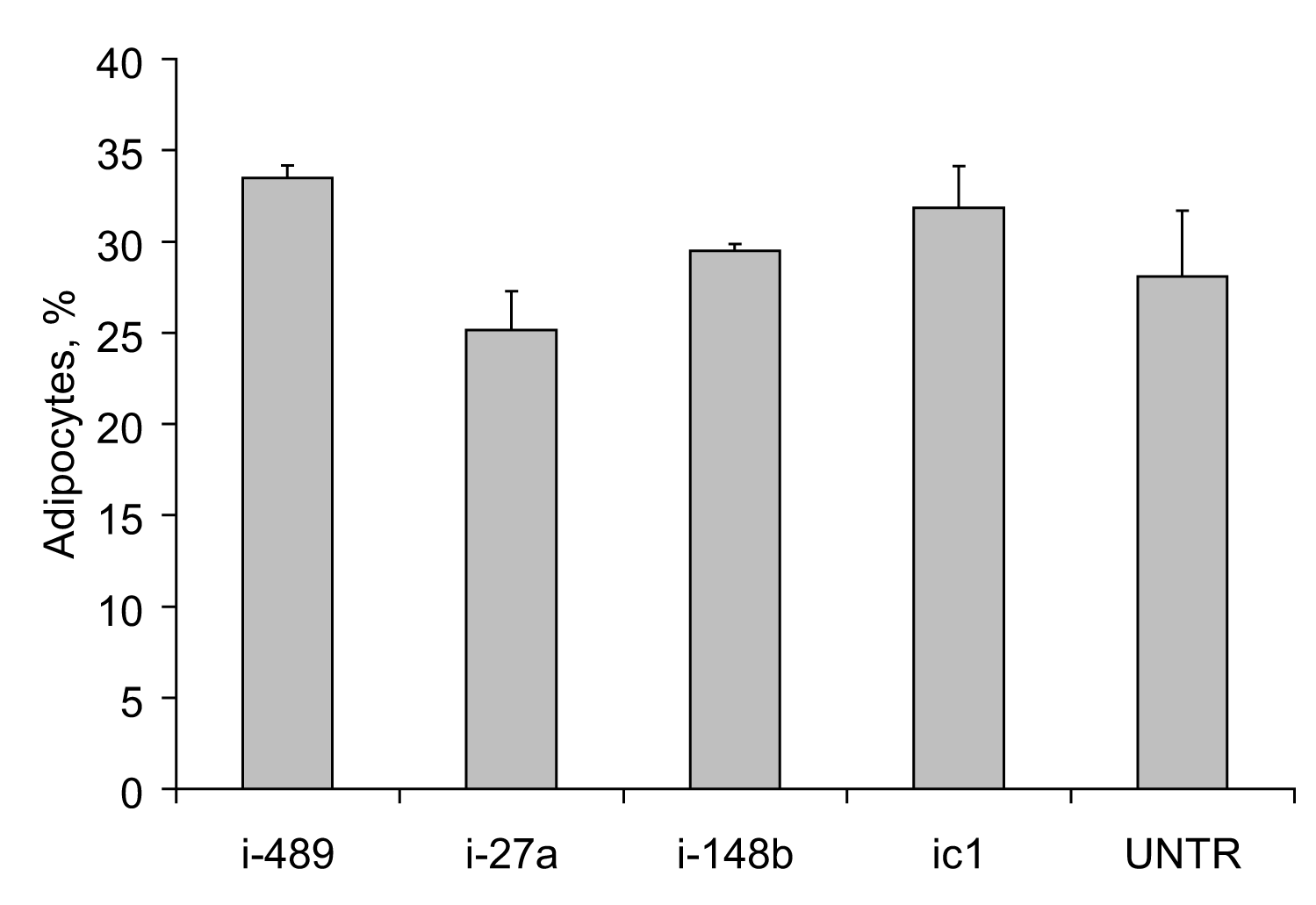

Supplement: Figure S2 — Inhibition of miR-148b, -27a and -489 does not influence adipogenesis in hMSC. hMSC were transfected with miRNA inhibitors or control molecules ( all at 25 nM). Transfected cells were switched to differentiation 24 hr after transfection. Adipogenesis was determined as a fraction (%) of adipocytes in cultures. Data are representative of three independent experiments performed in triplicate. (mean+/−stdev). (1.55 MB TIF) [file pone.0005605.s002.tif]

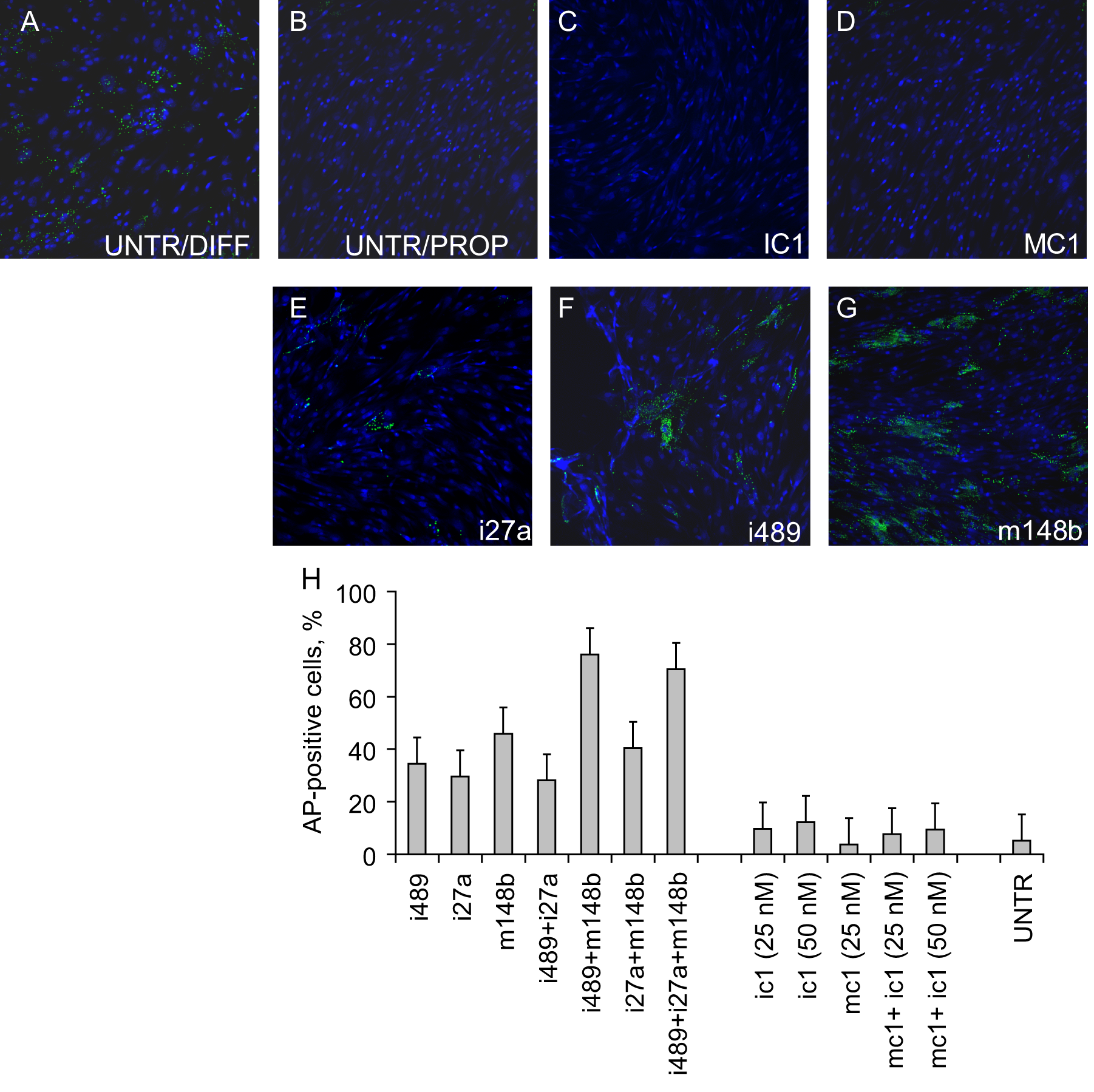

Supplement: Figure S3 — Alteration in miRNA activity results in increase of osteogenesis as measured by number of AP-positive cells. (A–G) Effect of alteration in miRNA activity results in increase of osteogenesis as measured by number of AP-positive cells in hMSC cultures. UNTR/Diff - untransfected differentiated cells, UNTR/Prop - untransfected undifferentiated cells, IC1,- cells transfected with Inhibitor Control Molecule 1, MC1,- cells transfected with Mimic Control Molecule 1, i27a - cells transfected with Inhibitor for miR27a, i489 - cells transfected with Inhibitor for miR489, m148b - cells transfected with mimic for miR148b. (H) Quantitative analysis of results obtained in experiments partially depicted in (A–F). (9.74 MB TIF) [file pone.0005605.s003.tif]

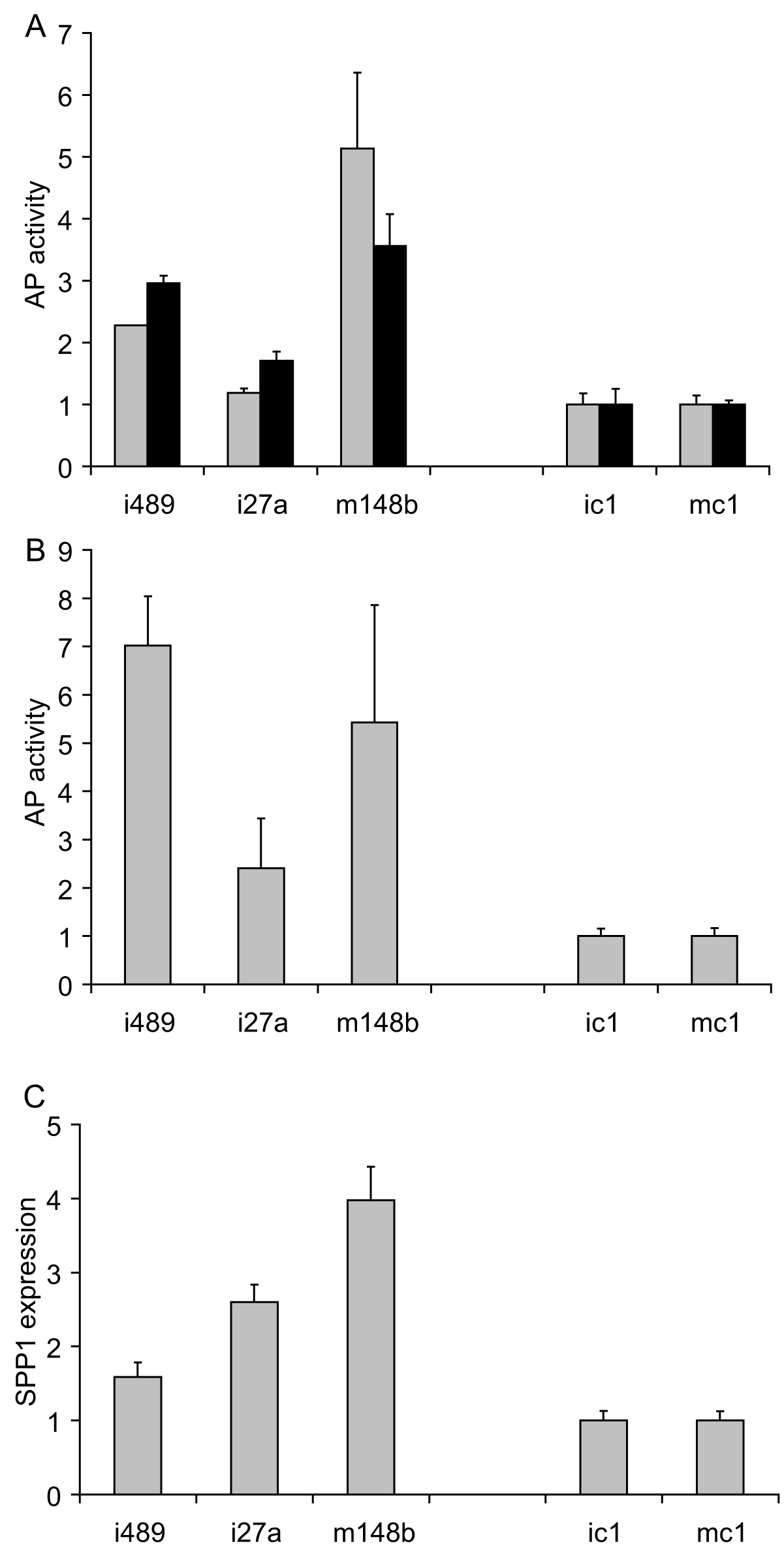

Supplement: Figure S4 — Alteration in miRNA activity produced similar results in hMSCs from two different donors and in human adult stem cells originated from adipose tissue. (A) Results obtained on AP activity of hMSCs from two different donors Alteration in miRNA activity in human adult stem cells derived from adipogenic tissue results in increase of AP activity (B) and up-regulation of SPP1 expression (C). Cells were treated as described in Supplemental Figure 2 legend are representative of three independent experiments performed in triplicate. (mean+/−stdev). (8.66 MB TIF) [file pone.0005605.s004.tif]

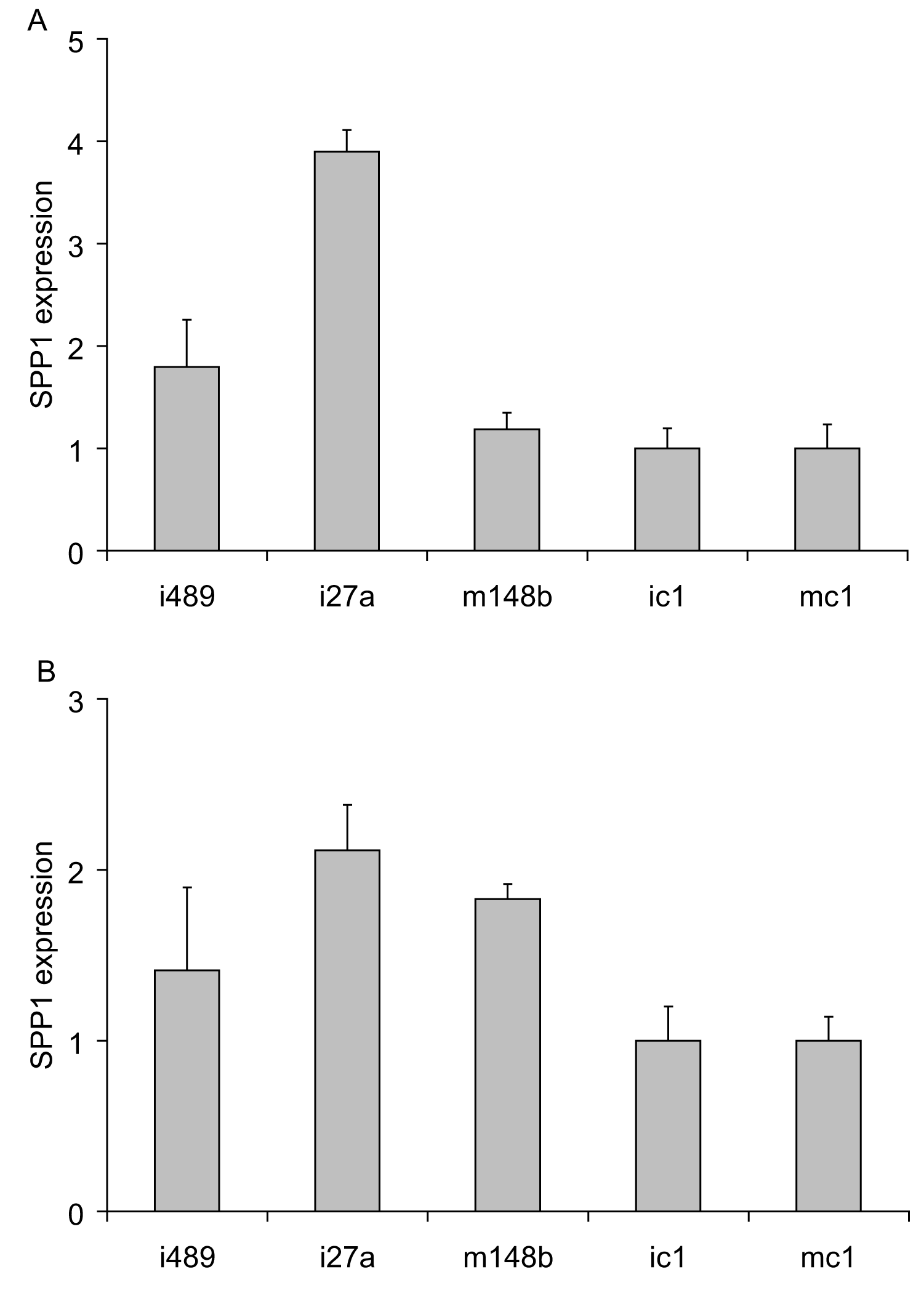

Supplement: Figure S5 — Alteration of the miRNAs activities transfection rescues osteogenic potential in transfected hMSC with high passage number. Transfection with miRNA inhibitors and mimics restores differentiation in overpropagated hMSC incubated in propagation (A) or differentiation (B) media as measured by SPP1 (osteopontin) expression. Data are representative of three independent experiments performed in triplicate. (mean+/−stdev). (3.16 MB TIF) [file pone.0005605.s005.tif]

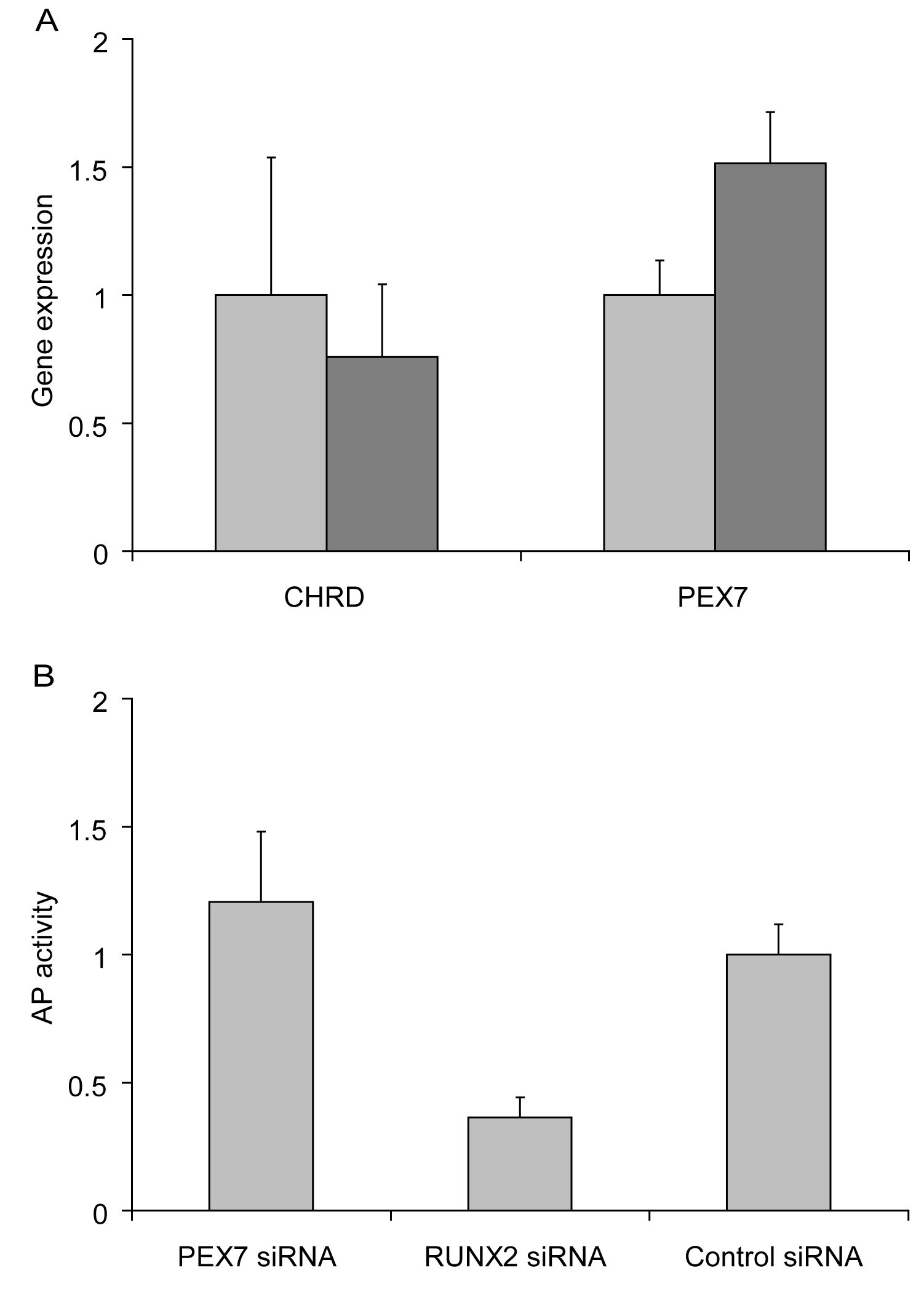

Supplement: Figure S6 — Effect of on osteogenesis miR-489 and -27a is not mediated by repression of CHRD or PEX7. (A) RT-PCR-based study of gene expression demonstrated that the expression of PEX7 but not CHRD mRNA is regulated by miR-489 and -27a. (A) siRNA-mediated knockdown of PEX7 did not produce a significant decrease of AP activity in hMSC under differentiation conditions. Data shown represents two independent transfection experiments performed in duplicate (A) or triplicate (B) (mean+/−SD). (3.16 MB TIF) [file pone.0005605.s006.tif]

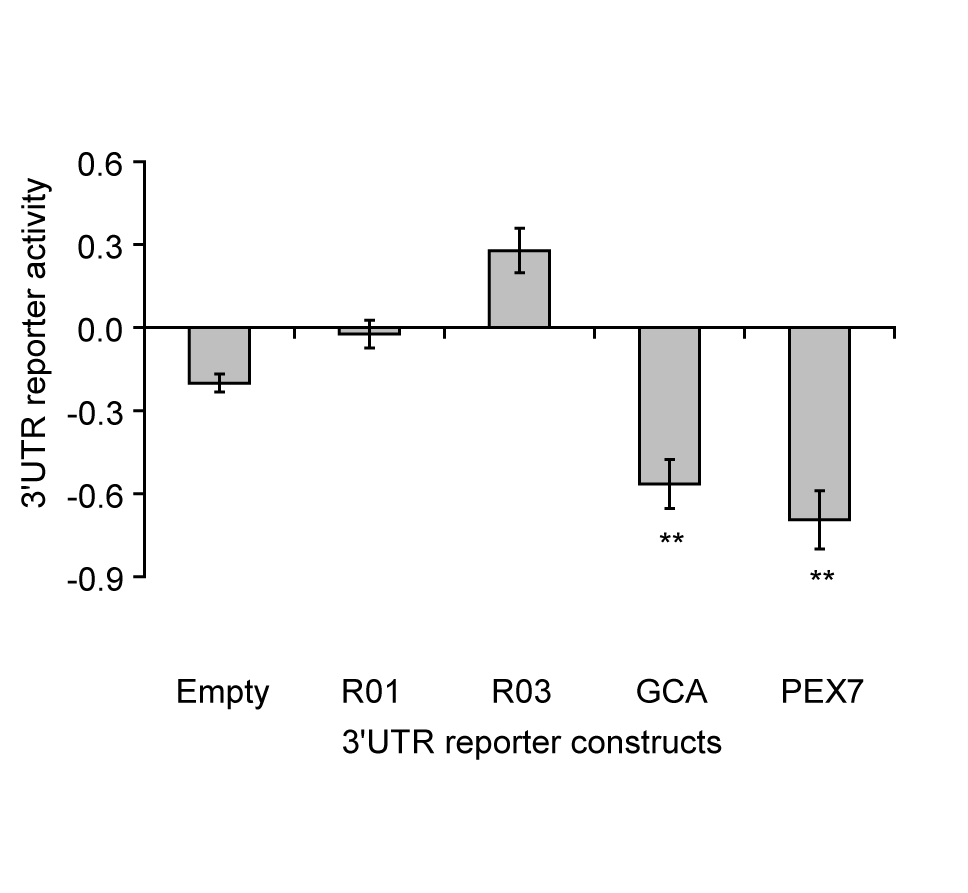

Supplement: Figure S7 — GCA and PEX7 3′UTR reporter activity is regulated by miR-489 and -27a. HT1080 cells were transfected with Mimic Control molecule 1 (mc1, 12.5 nM) or with a combination of inhibitors for miR-27 and -489 (i27a+i489, 6.25 nM each) and with 3′UTR reporter constructs as imdicated. Transfected cells were incubated for 48 and then harvested and processed as described in Materials and Methods. Data shown represents 3 individual transfections (mean+/−SD). **- p<0.05, Student's ttest p value between treated cells and corresponding control group. (0.87 MB TIF) [file pone.0005605.s007.tif]

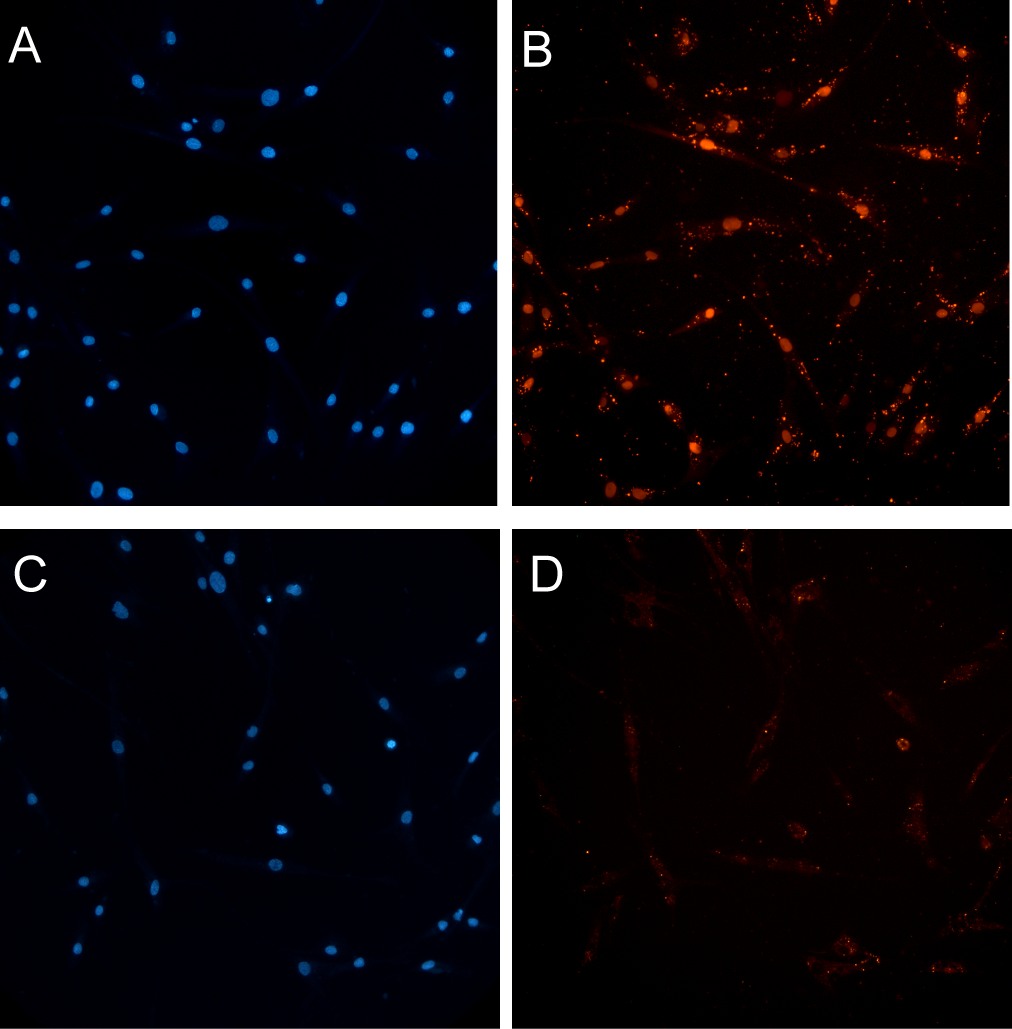

Supplement: Figure S8 — Uptake of fluorescently labeled Inhibitor Control Molecule 1 or Mimic Control Molecule 1 by hMSCs. Cells were transfected with Dy549-labeled MimicControl molecule 1 (C, D) or Inhibitor Control Molecule 1 (A,B) as described in Materials and Methods. Live cells were stained with Hoechst 33342 nuclear dye (A,C) 24 hr after transfection and photographed. (3.14 MB TIF) [file pone.0005605.s008.tif]

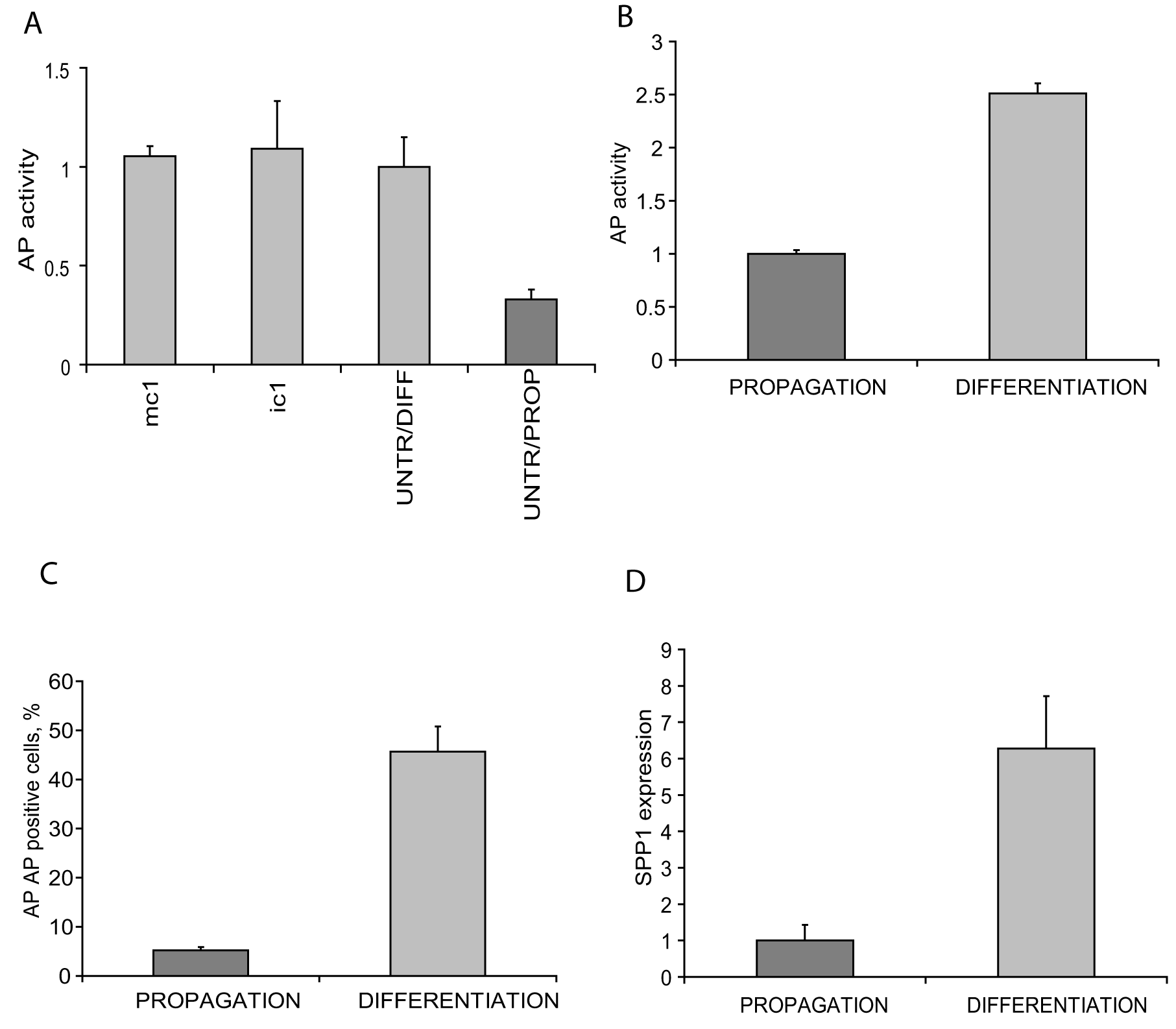

Supplement: Figure S9 — Osteogenic differentiation of hMSCs (A) Activation of AP in hMSCs incubated in osteogenic media for 6 days. Cells were incubated either in propagation media (Propagation) or in osteogenic media (Differentiation). (B) Increase in the number of AP-positive cells in differentiated cultures of hMSC harvested at 6 days of incubation in osteogenic media. Cells were incubated either in propagation media (Propagation) or in osteogenic media (Differentiation). (C) Upregulation of SPP1 expression in hMSCs incubated in osteogenic media for 6 days. Cells were incubated either in propagation media (Propagation) or in osteogenic media (Differentiation). (D) Effect of control miRNA inhibitor and mimic on differentiation of hMSCs. Cells were transfected with MimicControl molecule 1 (MC1) or Inhibitor Control Molecule 1 (IC1) as indicated. Transfected cells were switched to differentiation 24 hr after transfection. AP activity was measured in hMSCs incubated in osteogenic media for 6 days (MC1, IC1 and UNTR/Diff - untransfected control cells) or in untransfected cells incubated in propagation media (UNTR/Undiff) . Cells were incubated either in propagation media (Propagation) or in osteogenic media (Differentiation). Data are representative of three independent experiments performed in triplicate. (mean+/−stdev). (2.89 MB TIF) [file pone.0005605.s009.tif]
