# Supplementary material for: Functional Profiling Reveals Critical Role for miRNA in Differentiation of Human Mesenchymal Stem Cells
Source: PLoS One. 2009 May 19;4(5):e5605. doi: 10.1371/journal.pone.0005605 (PMC2680014; doi:10.1371/journal.pone.0005605)
Supplement: Table S1 — Expression of miR-148b, -27a and -489 in hMSCs under propagation and differentiation conditions. (0.05 MB DOC) [file pone.0005605.s010.doc]

| **miRNA** | **Conditions** | **Copy number per cell** | |  | **T-test P-value** |  |
| --- | --- | --- | --- | --- | --- | --- |
|  |  |  |  |  |  |  |
| hsa-mir27a | PROPAGATION | 90.93 |  |  | 0.009614 |  |
|  | OSTEOGENIC | 53.19 |  |  |  |  |
| hsa-mir148b | PROPAGATION | 14.09 |  |  | 0.016487 |  |
|  | OSTEOGENIC | 23.41 |  |  |  |  |
| has-mir489 | PROPAGATION | 0.906375 |  |  | 0.093047 |  |
|  | OSTEOGENIC | 0.089395 |  |  |  |  |
