# Supplementary material for: Functional Profiling Reveals Critical Role for miRNA in Differentiation of Human Mesenchymal Stem Cells
Source: PLoS One. 2009 May 19;4(5):e5605. doi: 10.1371/journal.pone.0005605 (PMC2680014; doi:10.1371/journal.pone.0005605)
Supplement: Table S2 — Genes that are predicted as a target for miR-148b, 27a and 489 and involved into skeletal development. (0.08 MB DOC) [file pone.0005605.s011.doc]

|  | **Gene Name** | **Description** | |
| --- | --- | --- | --- |
|  |  |  |  |
| miR-148b targets |  |  |  |
|  | NOG | noggin |  |
|  | HOXA5 | homeobox A5 | |
|  | CSF1 | colony stimulating factor 1 (macrophage) | |
|  | COL2A1 | collagen, type II, alpha 1 (primary osteoarthritis, spondyloepiphyseal dysplasia, congenital) | |
|  | TRPS1 | trichorhinophalangeal syndrome I | |
|  | ACVR1 | activin A receptor, type I | |
|  | FBN1 | fibrillin 1 |  |
|  | RUNX1 | runt-related transcription factor 1 (acute myeloid leukemia 1; aml1 oncogene) | |
|  |  |  |  |
|  |  |  |  |
| miR-27a targets |  |  |  |
|  | ADAMTS4 | ADAM metallopeptidase with thrombospondin type 1 motif, 4 | |
|  | AHSG | alpha-2-HS-glycoprotein | |
|  | ALPL | alkaline phosphatase, liver/bone/kidney | |
|  | BMI1 | BMI1 polycomb ring finger oncogene | |
|  | CHRD | chordin |  |
|  | DLX5 | distal-less homeobox 5 | |
|  | HOXA13 | homeobox A13 | |
|  | MATN1 | matrilin 1, cartilage matrix protein | |
|  | MINPP1 | multiple inositol polyphosphate histidine phosphatase, 1 | |
|  | PEX7 | peroxisomal biogenesis factor 7 | |
|  | PRKRA | protein kinase, interferon-inducible double stranded RNA dependent activator | |
|  | THRA | thyroid hormone receptor, alpha (erythroblastic leukemia viral (v-erb-a) oncogene homolog, avian) | |
|  |  |  |  |
| miR-489 targets |  |  |  |
|  | AHSG | alpha-2-HS-glycoprotein | |
|  | AMELY | amelogenin, Y-linked | |
|  | BMP7 | bone morphogenetic protein 7 (osteogenic protein 1) | |
|  | CDH11 | cadherin 11, type 2, OB-cadherin (osteoblast) | |
|  | CHRD | chordin |  |
|  | CHRDL2 | chordin-like 2 | |
|  | EN1 | engrailed homeobox 1 | |
|  | MAPK8 | mitogen-activated protein kinase 8 | |
|  | MGP | matrix Gla protein | |
|  | PAX1 | paired box gene 1 | |
|  | PEX7 | peroxisomal biogenesis factor 7 | |
|  | POSTN | periostin, osteoblast specific factor | |
|  | SPP1 | secreted phosphoprotein 1 (osteopontin, bone sialoprotein I, early T-lymphocyte activation 1) | |
|  | SUFU | suppressor of fused homolog (Drosophila) | |
|  | TRAPPC2 | trafficking protein particle complex 2 | |
|  | TWIST1 | twist homolog 1 (acrocephalosyndactyly 3; Saethre-Chotzen syndrome) (Drosophila) | |
