# Supplementary material for: Functional Profiling Reveals Critical Role for miRNA in Differentiation of Human Mesenchymal Stem Cells
Source: PLoS One. 2009 May 19;4(5):e5605. doi: 10.1371/journal.pone.0005605 (PMC2680014; doi:10.1371/journal.pone.0005605)
Supplement: Table S3 — Genes that are predicted as common targets for miR-27a and -489. (0.09 MB DOC) [file pone.0005605.s012.doc]

| **Gene Name** | **Description** |
| --- | --- |
| AHSG | Alpha-2-HS-glycoprotein |
| ALKBH3 | AlkB, alkylation repair homolog 3 (E. coli) |
| AMY2B | Amylase, alpha 2B (pancreatic) |
| ANXA5 | Annexin A5 |
| ASB3 | Ankyrin repeat and SOCS box-containing 3 |
| ATP5G3 | ATP synthase, H+ transporting, mitochondrial F0 complex, subunit C3 (subunit 9) |
| B4GALT3 | UDP-Gal:betaGlcNAc beta 1,4- galactosyltransferase, polypeptide 3 |
| BCOR | BCL6 co-repressor |
| BEX2 | Brain expressed X-linked 2 |
| BEX3_HUMAN | Brain expressed X-linked 3 |
| BYSL | Bystin-like |
| C10orf137 | Chromosome 10 open reading frame 137 |
| C3orf39 | Chromosome 3 open reading frame 39 |
| C6orf78 | Chromosome 6 open reading frame 78 |
| CHRD | Chordin |
| CI068_HUMAN |  |
| COX8A | Cytochrome c oxidase subunit 8A (ubiquitous) |
| CTSZ | Cathepsin Z |
| DISC1 | Disrupted in schizophrenia 1 |
| ELOVL1 | Elongation of very long chain fatty acids (FEN1/Elo2, SUR4/Elo3, yeast)-like 1 |
| EXOSC1 | Exosome component 1 |
| FCRL2 | Fc receptor-like 2 |
| FER1L3 | Fer-1-like 3, myoferlin (C. elegans) |
| GCA | Grancalcin, EF-hand calcium binding protein |
| GSPT1 | G1 to S phase transition 1 |
| GSPT2 | G1 to S phase transition 2 |
| HYDIN | hydrocephalus inducing homolog |
| IFNG | Interferon, gamma |
| JHD2C_HUMAN |  |
| KCNK1 | Potassium channel, subfamily K, member 1 |
| KIAA0146 | KIAA0146 |
| KYNU | Kynureninase (L-kynurenine hydrolase) |
| LAPTM4A | Lysosomal-associated protein transmembrane 4 alpha |
| MAGEC1 | Melanoma antigen family C, 1 |
| MB | Myoglobin |
| MLPH | Melanophilin |
| MLSTD1 | Male sterility domain containing 1 |
| MRPS23 | Mitochondrial ribosomal protein S23 |
| MTMR12 | myotubularin related protein 12 |
| NFIL3 | Nuclear factor, interleukin 3 regulated |
| NOX3 | NADPH oxidase 3 |
| NP_056174.1 | solute carrier family 39 (zinc transporter), member 14 isoform 2 |
| NP_116094.2 | hypothetical protein LOC84791 |
| NU6M_HUMAN |  |
| PBXIP1 | Pre-B-cell leukemia homeobox interacting protein 1 |
| PCM1 | Pericentriolar material 1 |
| PEBP1 | Phosphatidylethanolamine binding protein 1 |
| PEX7 | Peroxisomal biogenesis factor 7 |
| PMF1 | Polyamine-modulated factor 1 |
| PO210_HUMAN |  |
| PPA1 | Pyrophosphatase (inorganic) 1 |
| PPIF | Peptidylprolyl isomerase F (cyclophilin F) |
| PPIL4 | Peptidylprolyl isomerase (cyclophilin)-like 4 |
| PTCH1 | patched homolog |
| Q4V339_HUMAN |  |
| Q8TEB0_HUMAN |  |
| RAB3GAP1 | RAB3 GTPase activating protein subunit 1 (catalytic) |
| RBBP6 | Retinoblastoma binding protein 6 |
| RPS12 | Ribosomal protein S12 |
| RRAS | Related RAS viral (r-ras) oncogene homolog |
| RWDD2 |  |
| SASH1 | SAM and SH3 domain containing 1 |
| SLC22A2 | Solute carrier family 22 (organic cation transporter), member 2 |
| SPTAN1 | Spectrin, alpha, non-erythrocytic 1 (alpha-fodrin) |
| STOML2 | Stomatin (EPB72)-like 2 |
| TBC1D8B | TBC1 domain family, member 8B |
| TIP1_HUMAN |  |
| TLX1 | T-cell leukemia homeobox 1 |
| TMEM16K | Transmembrane protein 16K |
| TOP2B | Topoisomerase (DNA) II beta 180kDa |
| TPR | Translocated promoter region (to activated MET oncogene) |
| TRERF1 | Transcriptional regulating factor 1 |
| TSPAN1 | Tetraspanin 1 |
| UAP1 | UDP-N-acteylglucosamine pyrophosphorylase 1 |
| UBE2U | Ubiquitin-conjugating enzyme E2U (putative) |
| UGP2 | UDP-glucose pyrophosphorylase 2 |
| VP13B_HUMAN |  |
| ZBTB8_HUMAN |  |
| ZNF385C | zinc finger protein 385C |
