# Supplementary material for: Functional Profiling Reveals Critical Role for miRNA in Differentiation of Human Mesenchymal Stem Cells
Source: PLoS One. 2009 May 19;4(5):e5605. doi: 10.1371/journal.pone.0005605 (PMC2680014; doi:10.1371/journal.pone.0005605)
Supplement: Table S4 — Genomic coordinates of the fragments cloned into 3′UTR reporter constructs. (0.04 MB DOC) [file pone.0005605.s013.doc]

| **Vector** | **Genomic coordinates** |
| --- | --- |
| GCA 3'UTR vector | [chr2+: 163042229 - 163043204](http://genome.ucsc.edu/cgi-bin/hgTracks?clade=mammal&org=Human&db=hg17&position=chr2%3A163042229-163043204) |
| PEX7 3'UTR vector | chr6+: 137276293 - 137276787 |
| RO1 control vector | chr13+: 82190847 - 82193385 |
| RO3 control vector | chr13+: 82120892 - 82121673 |
